# Case report: Pellagra presentation with dermatitis and dysphagia

**DOI:** 10.3389/fmed.2024.1390180

**Published:** 2024-07-09

**Authors:** Segenet Bizuneh Mengistu, Iman Ali, Hiwot Alemu, Endalkachew Belayneh Melese

**Affiliations:** ^1^School of Medicine, College of Medicine and Health Science, University of Gondar, Gondar, Ethiopia; ^2^Johns Hopkins University School of Medicine, Baltimore, MD, United States

**Keywords:** pellagra, dermatitis, dysphagia, case report, global health

## Abstract

Pellagra is a disorder caused by a deficiency of niacin or tryptophan, manifested by characteristic dermatitis on sun-exposed areas of the skin. Gastrointestinal involvement is common, and symptoms include glossitis, stomatitis, and diarrhea. Neurologic symptoms can occur in some patients, including dementia, anxiety, depression, tremors, hyporeflexia and, in severe cases, encephalopathy. We present the case of a woman with hyperpigmentation and hyperkeratosis on sun-exposed areas of the skin along with progressive dysphagia. Notably, she did not report diarrhea or any neurologic or psychiatric symptoms. Her symptoms were most consistent with pellagra, and niacin supplementation was initiated, leading to recovery. This case report highlights that dermatitis and dysphagia, the main gastrointestinal manifestations, can be the only symptoms in patients with pellagra, requiring a high index of suspicion in dermatologic settings to diagnose and treat this fatal condition early.

## Introduction

First recognized by Gaspar Casal in 1735, pellagra remained a major widespread cause of death until the early 20th century (1). It is caused by a deficiency of niacin and manifests as the traditional triad of dermatitis, diarrhea, and dementia (2, 3). It has now been essentially eradicated in developed countries due to advancements in public health and nutrition through the enrichment of wheat flour with nicotinic acid; thus, only isolated cases are reported among patients with alcohol use disorders or carcinoid syndrome (4–7).

We present a rare case of pellagra in rural northern Ethiopia. The patient presented with photosensitive dermatitis and progressive dysphagia. Even though dermatitis and glossitis are common manifestations of pellagra, she did not have any other gastrointestinal or neurologic symptoms. This presentation of pellagra with predominantly dermatologic findings underscores the significance of considering it in the differential diagnosis in a dermatology setting, with careful consideration of the patient’s history and risk factors.

## Case description

**Figure 1:**
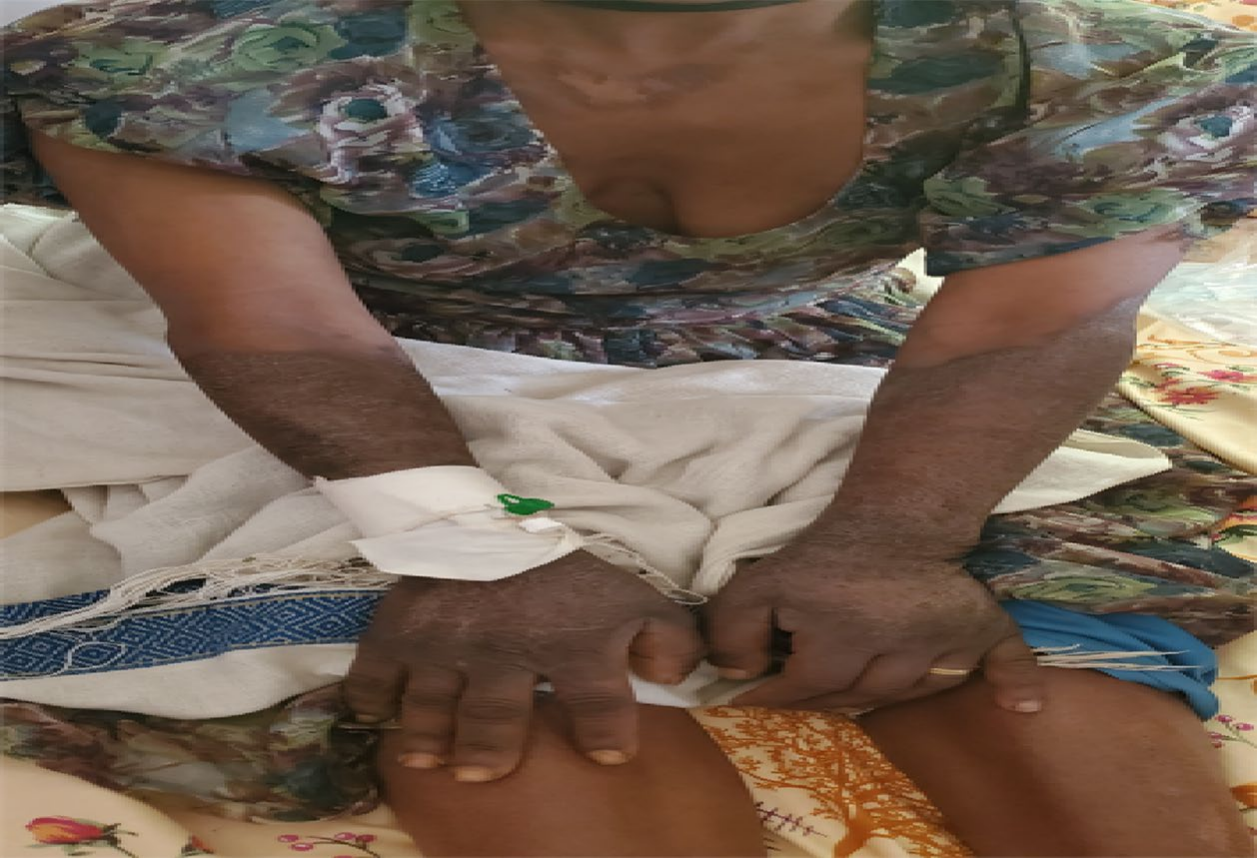
Dermatitis on bilateral forearms and dorsal hands at the time of presentation.

A 27-year-old woman without significant past medical history was presented to the University of Gondar Referral Hospital with a 2-year history of hyperpigmented and pruritic skin lesions over the neck, bilateral forearms, and dorsal surface of her hands that were exacerbated by sun exposure (Figure 1). During this period, she also reported progressively worsening difficulty swallowing solid foods. She had no history of diarrhea or any changes in her mental state or behavior. The patient did not report taking any medications, and she had no history of tuberculosis treatment. She reported occasionally drinking homemade alcohol. No one in her household or family had similar symptoms. Her diet consists mainly of maize and millet due to her lack of economic resources to consume other foods.

On examination, the patient had atrophied tongue papillae and well-demarcated, hyperpigmented, erythematous lichenified plaques involving her bilateral forearms, hands, and neck. The rest of her physical exam was unremarkable. Esophagogastroduodenoscopy revealed mild duodenitis without esophageal stenosis, erosion, or masses. Given her presentation, age, and gender, various autoimmune diseases, including systematic lupus erythematous, Sjogren’s, and rheumatoid arthritis, were considered. The basic laboratory workup, including a complete blood count, comprehensive metabolic panel, anti-nuclear antibody, rheumatoid factor, hepatitis B surface antigen, hepatitis C antibody, and HIV testing, were unremarkable (Table 1). The erythrocyte sedimentation rate was mildly elevated, and she tested positive for *Helicobacter pylori* antigen in her stool.

**Table 1:** Laboratory workup performed on the patient at the time of presentation.

	Value
White blood cell count	5,100 k/μL
Hemoglobin	15.8 g/dL
Platelet	282,000 k/μl
Na^+^	138 mEq/L
K^+^	4.4 mEq/L
BUN	10 mg/dL
Creatinine	0.54 mg/dL
Albumin	4.9 g/dL
AST	21 U/L
ALT	14 U/L
Random glucose level	82 mg/dL
HBsAg	Negative
HCV Ab	Negative
PITC^*^	Non-reactive
Anti-nuclear antibody	Non-reactive
Rheumatoid factor	Non-reactive
Erythrocyte sedimentation rate	23 mm/h
Stool *H. pylori* antigen	Positive

* Provider-initiated testing and counseling for human immunodeficiency virus.

Primary dietary niacin deficiency was suspected to be the most likely cause of her presentation, given that she is from a low socioeconomic background in rural Ethiopia, with her diet consisting mainly of maize and millet. We were unable to perform serologic or urinary niacin assays due to limited resources in our setting. Empiric treatment with multivitamins containing 50 mg of niacin three times per day orally was started. She was also advised to consume foods rich in niacin, such as meat, vegetables, and peanuts, when available. Within 2 weeks of starting treatment, she had a follow-up visit, and the dermatitis had improved significantly, as had her dysphagia. Overall, the patient reported significant improvements in symptoms and overall quality of life and was grateful to have received quality care after dealing with her symptoms for years. The patient was instructed to continue her multivitamin supplementation for an additional month. At the time of her follow-up visit, the patient was also initiated on *H. pylori* eradication treatment (Table 2).

**Table 2:** Timeline of care.

Initial visit	A comprehensive history of the present illness and physical examination performed
	Basic laboratory workup ordered
	Esophagogastroduodenoscopy ordered
First follow-up visit	Diagnosed with primary dietary niacin deficiency
	Instructed to take multivitamins containing 50 mg of niacin three times per day orally
	Second follow-up visit scheduled in 2 weeks
Second follow-up visit	Marked improvement in presenting symptoms
	Started on *Helicobacter pylori* eradication therapy

## Discussion

Pellagra is a multisystem disorder due to a deficiency of niacin and the essential amino acid tryptophan. It can occur within 60 days of dietary niacin deficiency and is traditionally remembered as the “disease of the three Ds”: dermatitis, diarrhea, and dementia, and, if left untreated, death (1).

Based on the patient’s history of photosensitive dermatitis and dysphagia, as well as nutritional deficiency, primary pellagra caused by dietary niacin deficiency was suspected and confirmed with the resolution of symptoms following niacin administration. Secondary causes of pellagra include alcohol use disorder, iron deficiency anemia, malabsorption, and certain medications such as isoniazid, 6-mercaptopurine, 5-fluorouracil, azathioprine, anticonvulsants, and chloramphenicol (8, 9). Our patient has no history of alcohol use disorder, diarrhea, malabsorption, or prior medication use. Pellagra may also result from abnormalities of tryptophan metabolism, as seen in carcinoid syndrome, where tumor cells divert tryptophan metabolism toward serotonin and away from nicotinic acid. However, the absence of skin flushing, diarrhea, and wheezing on examination and her younger age make carcinoid syndrome unlikely. Hartnup disease is a congenital disability in tryptophan absorption and transfer and can also cause pellagra (10, 11). The presentation of her symptoms in adulthood and no family history of similar illnesses make Hartnup disease an unlikely cause of her presentation.

Dermatitis is the most characteristic finding of pellagra and usually commences as pruritic erythema in sun-exposed areas, particularly the dorsum of the hands, face, and neck (12, 13). This is followed by severe hyperpigmentation and hyperkeratosis, along with acute margination and epithelial desquamation. The characteristic Casal’s necklace of pellagra, which is hyperpigmentation around the neck extending to the chest, was present in our patient, which further aided in making an accurate diagnosis.

Dysphagia is a less common feature of pellagra than dermatitis but has been reported in up to 60% of patients and is attributed to inflammation of the oral mucosa (12, 14–16). Although pellagra is known as the “disease of the three Ds,” a small study found that only 22% of patients had the complete triad of dermatitis, diarrhea, and dementia, with diarrhea alone occurring in only 17% of patients and dementia in 28% (17).

The strength of this study is that we demonstrated that a patient with a predominantly dermatologic presentation of pellagra was promptly assessed and provided appropriate treatment for this life-threatening illness. To the best of our knowledge, a presentation for pellagra like our patient’s is uncommon and has not been reported. The main limitation of this study is that we were unable to quantitatively determine niacin levels due to the tests being unavailable at our limited-resource institution. In addition, we were not able to obtain collateral information on whether she had any neurologic or psychiatric symptoms during her illness. However, she denied these symptoms at presentation, and our mental and neurologic exams were unremarkable. Furthermore, we were unable to obtain images of her dermatitis post-treatment as she was lost to follow-up.

Overall, this case report demonstrates that pellagra can present with predominantly dermatologic symptoms, requiring a high degree of suspicion, especially in dermatologic settings, to accurately and promptly diagnose and manage this fatal condition.

## Data availability statement

The original contributions presented in the study are included in the article/supplementary material, further inquiries can be directed to the corresponding author.

## Ethics statement

Ethical approval was not required for the studies involving humans because case reports do not require ethical committee approval as long as informed consent is obtained. The studies were conducted in accordance with the local legislation and institutional requirements. The participants provided their written informed consent to participate in this study. Written informed consent was obtained from the individual(s) for the publication of any potentially identifiable images or data included in this article.

## Author contributions

SM: Conceptualization, Writing – original draft, Writing – review & editing. IA: Writing – original draft, Writing – review & editing. HA: Writing – original draft, Writing – review & editing. EM: Conceptualization, Writing – original draft, Writing – review & editing.
